# Challenges and opportunities for *Moringa* growers in southern Ethiopia and Kenya

**DOI:** 10.1371/journal.pone.0187651

**Published:** 2017-11-09

**Authors:** Diriba B. Kumssa, Edward J. M. Joy, Scott D. Young, David W. Odee, E. Louise Ander, Charles Magare, James Gitu, Martin R. Broadley

**Affiliations:** 1 School of Biosciences, University of Nottingham, Sutton Bonington, Loughborough, United Kingdom; 2 Centre for Environmental Geochemistry, British Geological Survey, Keyworth, Nottingham, United Kingdom; 3 Crops For the Future, The University of Nottingham Malaysia Campus, Jalan Broga, Semenyih, Selangor Darul Ehsan, Malaysia; 4 Faculty of Epidemiology and Population Health, London School of Hygiene & Tropical Medicine, London, United Kingdom; 5 Kenya Forestry Research Institute, Nairobi, Kenya; 6 Centre for Ecology and Hydrology, Bush Estate, Penicuik, Midlothian, United Kingdom; CSIR-Foood Research Institute, GHANA

## Abstract

*Moringa oleifera* (MO) and *M*. *stenopetala* (MS) are two commonly cultivated species of the Moringaceae family. Some households in southern Ethiopia (S. ETH) and Kenya (KEN) plant MS and MO, respectively. The edible parts of these species are rich in amino acids, vitamins and minerals, especially selenium. Despite their nutritional value, *Moringa* is sometimes considered as a “famine food”. The aim of this study was to determine the extent of dietary utilization of these plants by *Moringa* Growing Households (MGHs). *Moringa* growing households were surveyed in 2015. Twenty-four and 56 heads of MGHs from S. ETH and KEN, respectively, were interviewed using semi-structured questionnaires. Subsistence agriculture was the main source of livelihood for all MGHs in S. ETH and 71% of those in KEN. All MGHs in S. ETH cultivated MS while those in KEN cultivated MO. Of the MGH heads in S. ETH, 71% had grown MS as long as they remember; the median cultivation period of MO in KEN was 15 years. All MGHs in S. ETH and 79% in KEN used *Moringa* leaves as a source of food. Forms of consumption of leaves were boiled fresh leaves, and leaf powder used in tea or mixed with other dishes. Other uses of *Moringa* include as medicine, fodder, shade, agroforestry, and as a source of income. Although MO and MS have multiple uses, MGHs face several challenges, including a lack of reliable information on nutritional and medicinal values, inadequate access to markets for their products, and pest and disease stresses to their plants. Research and development to address these challenges and to promote the use of these species in the fight against hidden hunger are necessary.

## Introduction

Consumption of diverse diets, with balanced supplies of macro and micro-nutrients is required for normal human growth and physiological development. However, availability of optimally diverse diets may be constrained by wealth and/or education (including loss of traditional knowledge of indigenous crops). Human diets have been inadvertently simplified in food systems during the Green-Revolution era [1, 2], where agricultural production focused on provision of sufficient energy. In populations depending on cereal-based diets with low nutrient density, dietary simplification and shortage of access to animal source food exacerbates deficiency of vitamins and minerals, also known as hidden hunger [3–5]. *Moringa oleifera* (MO) *and M*. *stenopetala* (MS) are underutilized tropical tree species that can play an important role in dietary diversification and contribute to alleviation of hidden hunger in less developed tropical and subtropical countries [6–9]. In particular, *Moringa* can be a rich source of some micronutrients that are commonly deficient in cereal-based diets, e.g. selenium [6].

*Moringa oleifera* and MS are the two widely cultivated species of the Moringaceae family, which comprises 13 species. Previous ethnobotanical and biochemical studies in countries where *Moringa* is grown show that these species are multipurpose [10–13]. Various tissues are used as food, herbal medicine, fodder, hedges, firewood, gum and for water purification [14–22]. The foliage, immature pods, seeds, roots and young shoots are used as food and herbal medicine [16, 23]. *Moringa stenopetala* leaves are used in a similar way as cabbage and spinach and the tree is nicknamed the ‘cabbage tree’ [24]. Fresh MO and MS leaves are either boiled or consumed raw as vegetables, and leaf powders are mixed with other staple foods to increase the mineral, amino acid and vitamin density in the diets [6–8, 10, 16, 18, 23–26].

Despite their nutritious edible parts, *Moringa* spp. are sometimes classified as “famine food”, consumed by humans at times of food scarcity [24, 27, 28]. Similarly, preliminary information indicates that the human dietary usage of the edible parts of these species is limited. For example, in southern Ethiopia (S. ETH), *Moringa* tends to be cultivated by communities living in marginal environments, with small land holdings due to high population density [6]. In these areas, there is often a reliance on rain-fed agriculture as a source of livelihood and there are frequent food crop failures due to drought [12, 29]. The use of indigenous and locally available vegetables such as *Moringa* as a human food is often linked with low social class status in many communities in Africa and Asia [12, 15, 30, 31].

Few studies have assessed the ethnobotany of MO and MS, and these have focused primarily on their medicinal uses [8, 16, 17, 32–37]. There is a lack of information on the ethnobotany of MO and MS with emphasis on its use as human food source in S. ETH and Kenya (KEN). The aim of this study was to assess the current extent of dietary utilization of *Moringa* edible parts by *Moringa* Growing Households (MGHs) in S. ETH and KEN. This will help to identify where challenges and opportunities exist to widen the use of *Moringa* and reduce human mineral micronutrient deficiencies.

## Materials and methods

A questionnaire-based survey to assess the uses of MO and MS was conducted in S. ETH in April 2015 and various localities of KEN in July 2015. Prior to conducting the survey, research ethics approval was obtained from the University of Nottingham, School of Biosciences Research Ethics Committee (SB REC), approval number: SBREC140117A. A purposive sampling approach was pursued by identifying households that cultivated MO and MS, with emphasis on their utilization as a dietary source for human beings. Staff of the Kenyan Forestry Research Institute in KEN and an agricultural expert working for a local Non-Governmental Organization in S. ETH assisted to identify and select MGHs and to translate the questionnaire to local languages during the interviews.

A semi-structured interview was conducted with the MGH heads. For each participant, an information sheet (S1 Appendix) explaining the purposes of the survey, with details of the conditions of the interview and the rights of the interviewee were provided prior to the commencement of the interview. Respondents provided their consent (S2 Appendix) either by signature or thumb impression print and the questionnaire (S3 or S4 Appendices) was administered after obtaining the MGH head’s consent. The survey in Kenya was shortly after that in Ethiopia, and was integrated with the work of Kenya Forestry Research Institute who collected extra data on the experience in, and associated challenges of *Moringa* cultivation by MGH. Thus this additional information is only reported for Kenya MGH participants in this study. The study was carried out on private/communal land with the owners’ permission, and it did not involve endangered or protected species. Data collection was carried out using an online data collection system in KoboToolBox (http://www.kobotoolbox.org) using handheld mobile devices. When mobile data connection was unavailable in the field, the KoboToolBox saved the data temporarily on the device and uploaded it to a cloud server once connection to the Internet was re-established. A total of 24 and 56 MGH heads were interviewed in S. ETH and KEN, respectively. Subsequent statistical analysis and visualization was carried out using KoboToolBox and Tableau Desktop Professional Edition 10.

## Results

Summaries of the responses of MGH heads from S. ETH and KEN with regards to general household characteristics, cultivation of *Moringa* and challenges faced, and the dietary and other modes of utilization of *Moringa* are presented below.

### Southern Ethiopia

#### Household characteristics

**Fig 1:**
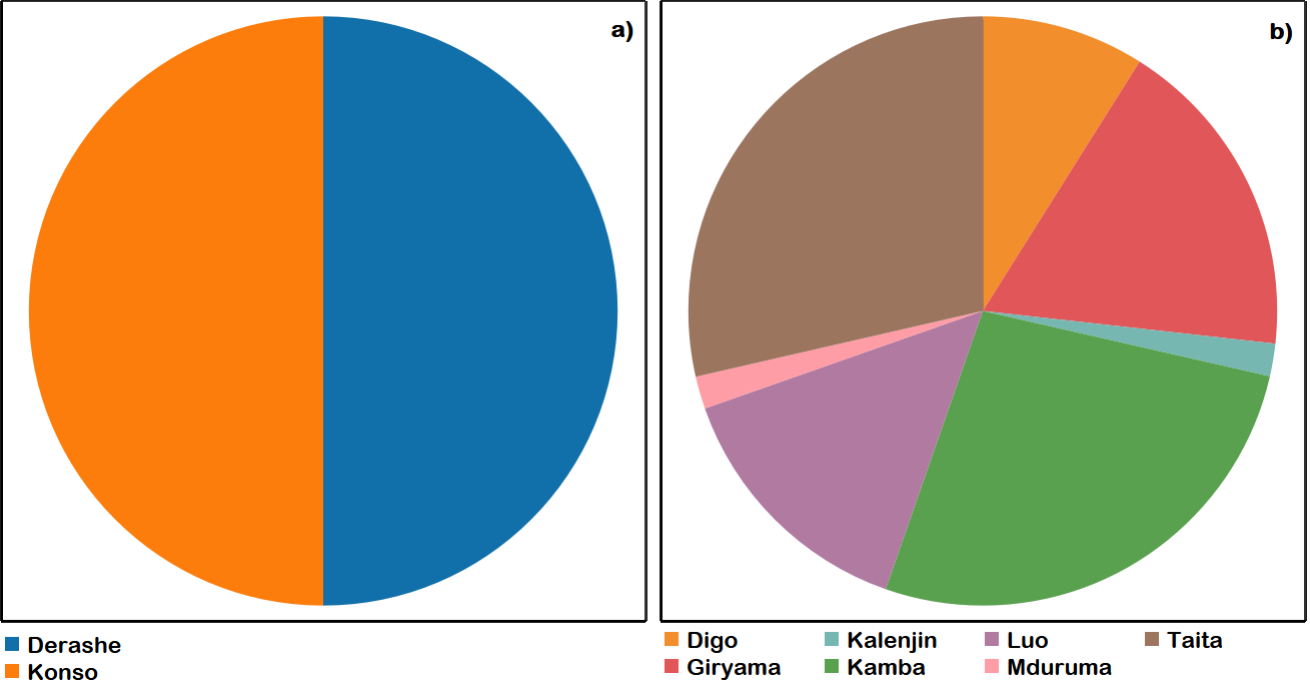
Ethnic groups to which the *Moringa* growing households belonged. Southern Ethiopia (a) and Kenya (b). Total number of respondents in southern Ethiopia (n = 24) and Kenya (n = 56).

The MGHs in S. ETH were from the Derashe and Konso ethnic groups (Fig 1). All the households (n = 24) grew MS and 75% of household heads were men, all of whom were married The median age of the MGH heads was 40 yrs and median number of fulltime residents of MGHs was 6 persons (Table 1). Fifty-eight percent of the MGH heads were illiterate (). The roof of the residential houses of 79% and 21% of the households were made from thatched grass and corrugated iron sheets, respectively. The floors of the residential houses of 96% and 4% of the households were earthen and cemented, respectively. None of the MGHs had electricity power supply or tap water at their residential houses. Potable water was obtained from boreholes (67%) and springs (33%). All MGHs relied on subsistence agriculture as sources of livelihood.

**Table 1:** Marital status, age, and educational level of *Moringa* growing household heads; the number of fulltime residents in the *Moringa* growing household; and the number of years for which *Moringa* has been cultivated; in southern Ethiopia (n = 24) and Kenya (n = 56).

Marital status (%)	S. ETH	KEN
Married	75	96
Single	8	4
Widowed	17	
**Age (yrs)**		
Mean	41	57
Standard deviation	8	13
**Educational level (%)**		
Illiterate	58	20
Elementary	25	48
High school	17	20
College	0	13
**Number of fulltime residents**		
Mean	6	6
Standard deviation	3	2
**Number of yrs of growing *Moringa***		
Mean		17
Standard deviation		12

#### Purposes of growing *M*. *stenopetala*

**Fig 2:**
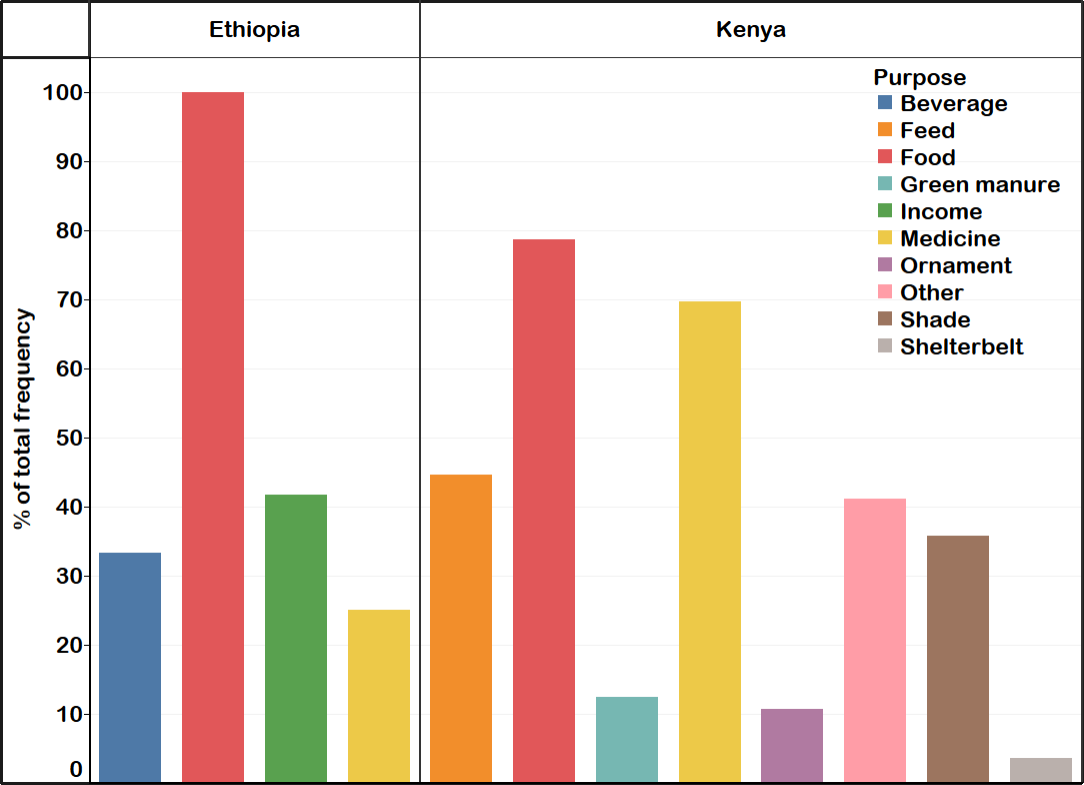
Purposes for which *Moringa* was grown in southern Ethiopia and Kenya. Number of respondents: southern Ethiopia (n = 24) and Kenya (n = 56).

**Fig 3:**
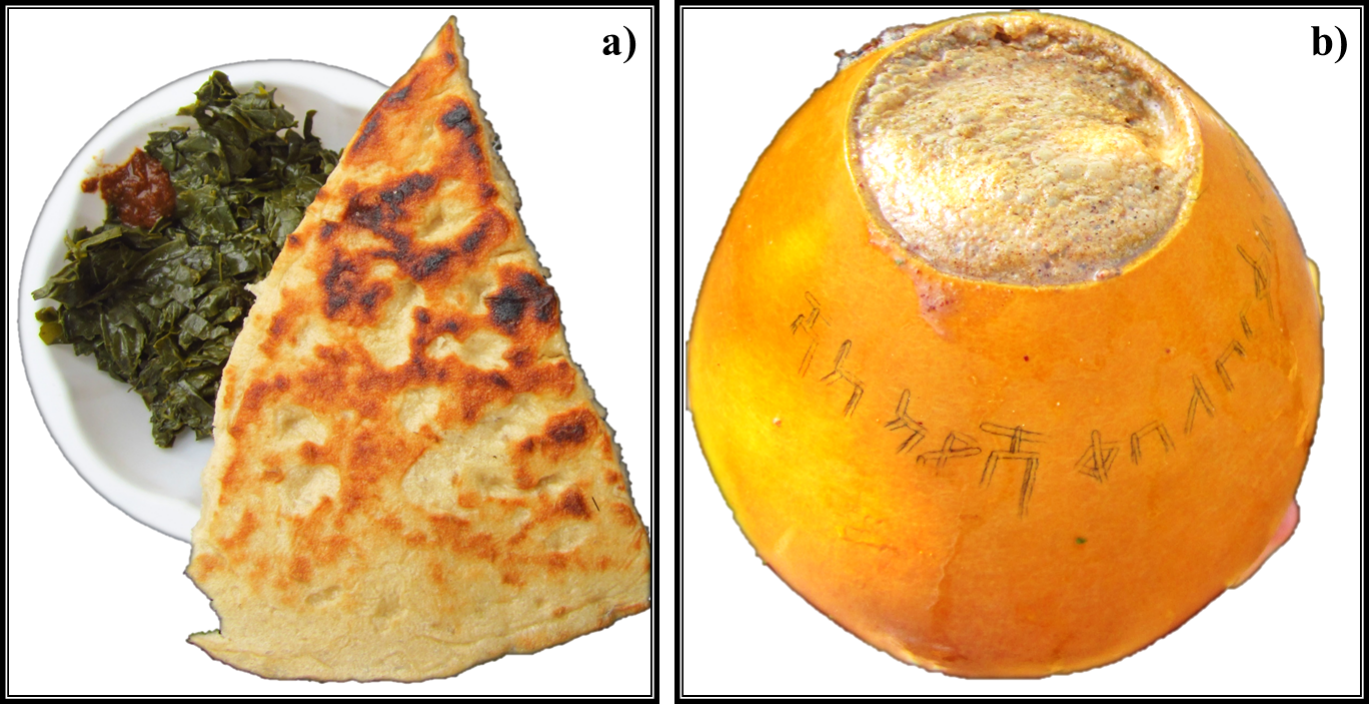
Commonly consumed foods, Karat Konso, southern Ethiopia. A typical breakfast of boiled *M*. *stenopetala* leaves with bread (a) and Chegga brewed from sorghum in a calabash gourd (b).

Seventy-one percent of the MGHs had been growing MS as long as they remember. The remaining 29% of the households had grown MS for 2–17 yrs. All MGHs had used MS as a source of food (Fig 2), with some also as a source of food and income (42%), as a source of food, income and drink (29%), and as source of food, drink and medicine (21%). *Moringa* growing households consumed boiled fresh leaves at a frequency of three times a day (92%) and most days in a week (8%). The quantity of leaves consumed per day were two big and medium bunches (4% each), one big bunch (42%), one medium bunch (29%) and one small bunch (21%). Other forms of consumption of MS included boiled flowers and immature pods, dried and crushed leaves mixed with traditional beverage made from sorghum (chegga) (Fig 3). While all the households from the Konso ethnic group consumed boiled fresh leaves of MS, those from the Derashe ethnic group consumed boiled fresh leaves (Fig 3), flowers and young pods, and dried and crushed leaf powder to make tea or mix it with chegga. Fifty percent of the MGHs from the Derashe ethnic group reported to have used MS as medicine in the following forms: fresh roots of the tree were crushed and inhaled to treat common cold; branches were broken to initiate sap outflow which was used as eye drops to treat eye infections; and fresh leaf juice had been used to treat head lice. Only one MGH head from the Konso ethnic group stated the use of MS as medicine, where the juices from fresh leaves were used to treat gastrointestinal parasites in cattle.

### Kenya

#### Household characteristics

All the households in KEN planted MO. The respondents were members of seven ethnic groups (Fig 1). Seventy percent and 30% of the MGH heads were men and women, respectively. Ninety-six percent of the MGH heads were married. Median age of the MGH head was 59 yrs and the median number of fulltime residents in a MGH was 6 (Table 1). In terms of the MGH head educational level, 48% had attended elementary school (Table 1). The land tenure was private (84%), communal (13%) and other (3%). Land holdings were 0.4–1.2 ha (68%), 1.3–4 ha (30%), and 4.1–6 ha (2%). Seventy-one percent of the households depend on subsistence agriculture as the source of their livelihood. Potable water sources of the MGHs were tap water (45%), river (32%), borehole (9%) and lake (7%). Roofs of the residential houses were made from corrugated iron sheets (86%), grass thatch (3%) and other (11%). Floors of the MGH residential houses were earthen (55%), cemented (43%) and tiled (2%). Only 16% of the MGHs had access to electricity power supply at their residential houses.

#### Purposes of growing *M*. *oleifera*

The period for which the MGH heads had been cultivating MO in various parts of KEN ranged between 1–59 yrs (Table 1). Planting of MO was conducted by direct seeding (84%), cuttings (11%) and seedlings (5%). Cuttings and seeds were obtained from neighbours (73%) who already had established MO trees while seedlings were purchased from nearby Department of Agriculture nurseries and Kenya Forestry Research Institute research stations. Nine percent of the respondents reported that a private company which promised to buy MO leaves, immature pods and seeds from farmers had distributed seedlings to some MGHs but did not fulfil the promise.

**Fig 4:**
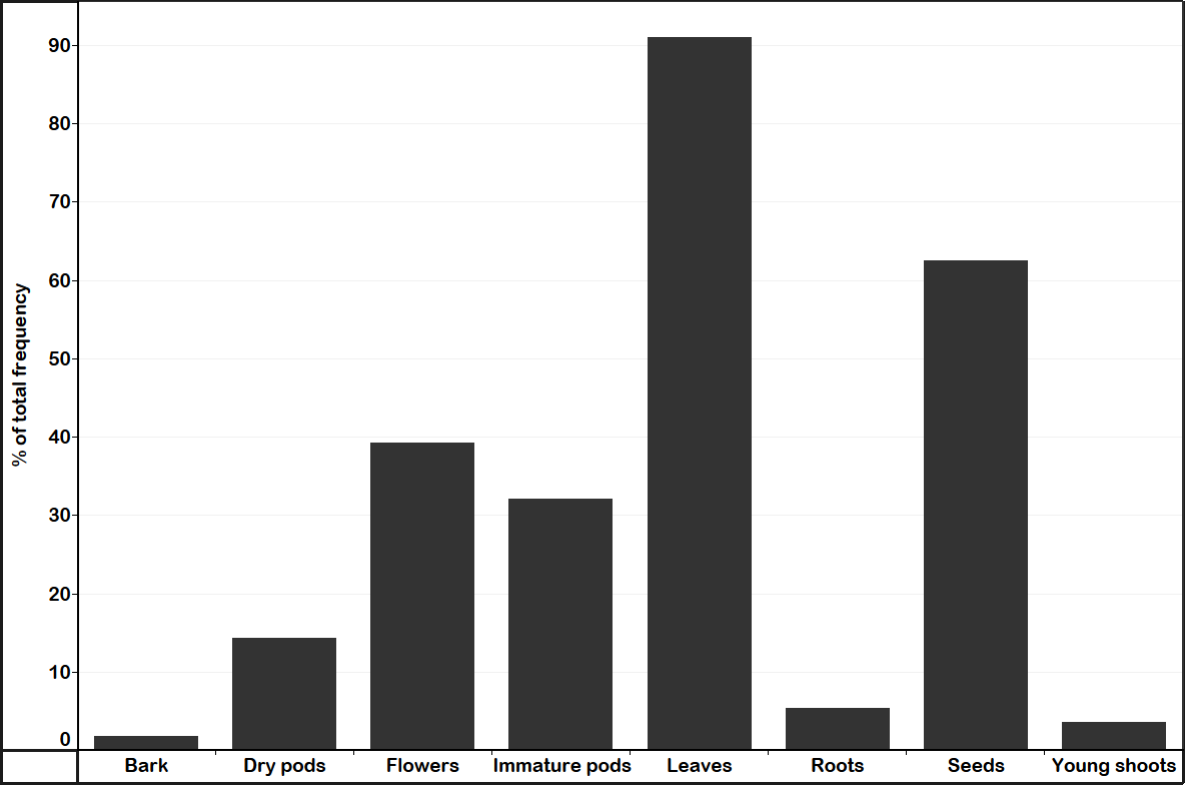
Parts of *M*. *oleifera* used by *Moringa* growing households in Kenya (n = 56).

The three main purposes for which the MGHs plant MO were food, medicine, and feed (Fig 2). Those MGHs that did not cultivate MO as a food source planted it for feed, medicine, shade, agroforestry, shelterbelt or other purposes. Many of the MGHs cultivated MO for multiple uses. Thirty-two percent of the MGHs cultivated MO for food, medicine, and feed; while 20% of them had been cultivating MO for food, feed, medicine and shade. The MO plant parts used for food, feed or medicine is indicated in Fig 4.

Among the respondents reporting that MO edible parts were used as food, 57% used the fresh leaves as a vegetable, and the remaining 22% used leaves as tea and leaf powders mixed with other foods, and young shoots and fresh flowers as vegetables. Some respondents reported that MO flowers tasted like eggs when fried with oil. Reported medicinal uses included: MO bark and roots boiled in water and the solution used to wash body and legs of diabetic patients to treat numbness and tingling sensations; leaves mixed with other foods or used as tea to treat high blood pressure, joint and general body pain, ulcers, food poisoning, and stomach problems. Some interviewees stated that the leaves, immature pods and seeds of MO were sold either for export or local markets and used as sources of income. Leaves, immature and mature pods were used as a source of feed mainly for goats.

#### Challenges in cultivation and use of *M*. *oleifera*

**Fig 5:**
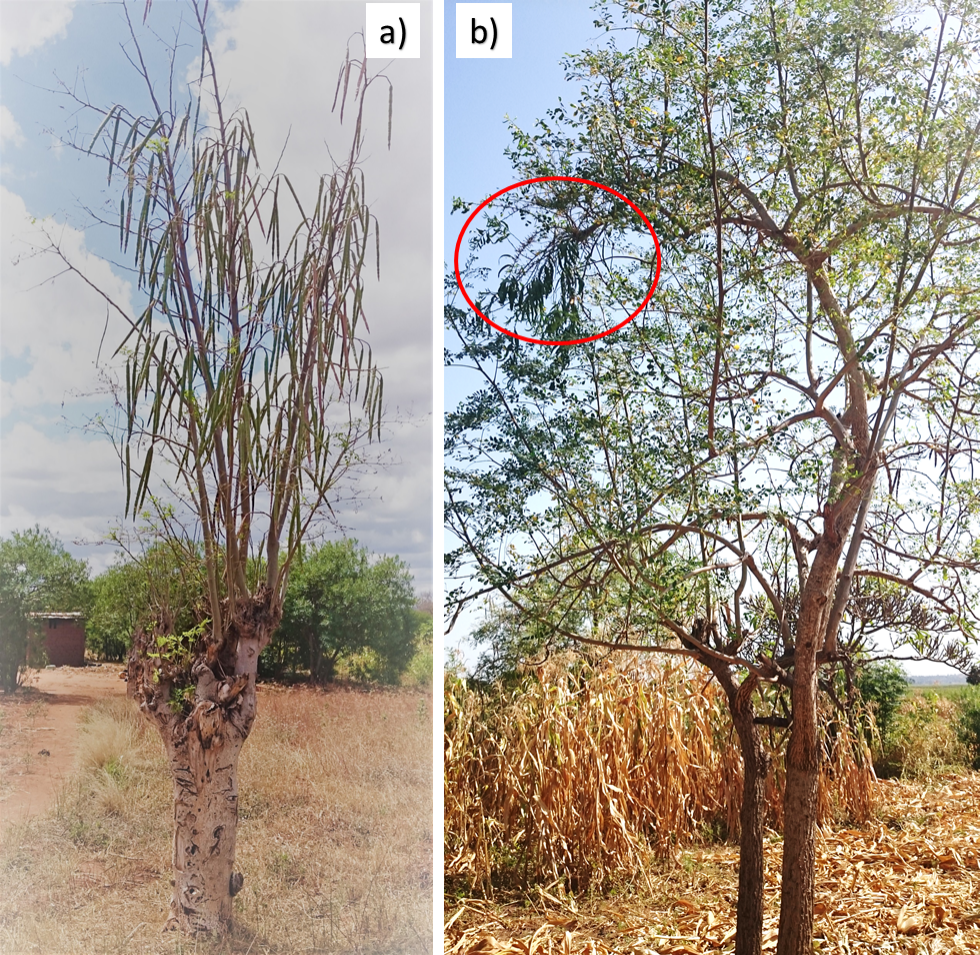
Pollarded *M*. *oleifera* tree in full pod at Kibwezi (a) and *M*. *oleifera* intercropped with maize at Ramogi (b) with parasitic plant growing on the branch (circled red), Kenya.

Eighty-four percent of the MGHs stated that they had encountered some challenges during the cultivation of MO. These include: pests and diseases (82%); rotting of trees grown on lands vulnerable to flooding; parasitic plants (Fig 5); low demand for MO products (7%); unknown dosage of MO edible parts used as medicine; uncertainty about the nutritional and medicinal values of MO.

Pest attacks have been reported during dry spells (50%), at the onset of the rainy season (32%) and all year round (2%). Insect larva mostly fed on the leaves (79%) and sometimes bored into the pods (18%). Larva foraging on the leaves reduced leaf biomass production and damaged leaves are unappealing for use as human food. Seeds in the pods bored by larva were damaged and became unviable for seeding and seedling production, and for other uses. Parasitic plants growing on MO were also observed in the field and reported as a problem by some MGH heads. The MGH heads were keen for advice on ways to get rid of diseases and pests that hinder the productivity and usability of the trees they cultivate. In areas that experience flooding in the rainy season, MO trees rot and die due to waterlogging, showing that MO prefers well drained soils [23].

Another challenge facing MGHs was the unclear and unreliable evidence regarding the medicinal and nutritional values of MO. They seek reliable evidence in regards to the uses of MO. Besides, for better access to secure markets, MGHs want support from government development and extension agents in providing education on the uses of MO to people who do not currently grow it. In KEN, MGH heads were asked whether they would like to obtain further information about MO. Eighty-six percent of the respondents wanted to get information about the medicinal and the nutritional values, for example, mineral micronutrients in edible parts of MO.

#### Tree management

The planting arrangements and management of the MO and MS trees were noted during the study. In S. ETH, trees were generally found in sorghum/maize fields, both on flat silty soils in Derashe and the sandy upland soils of the Konso terraces but some were found in household compounds. Coppicing was practiced widely with trees cut back in the early rainy season, although some were not coppiced. Tree coppicing is conducted to control the height growth (i.e., to keep leafy growth within reach) and to reduce shading when MS trees were intercropped with sorghum and maize. In KEN, MO trees were scattered within homesteads, as fences and hedges around homesteads, woodlots, and intercropped with staple crops such as, cassava, maize and sorghum. The most common method of tree management was pollarding (i.e., cutting back the canopy/branches of the tree) (Fig 5A). Some households practiced coppicing and lopping on the trees they cultivated. Weeding was conducted by some households who intercropped MO with maize and sorghum.

## Discussion

### On farm uses and services of *Moringa*

*Moringa oleifera* and MS produce nutritious flowers, leaves, and immature pods that can be used as human food and livestock fodder [13, 38–46]. Several studies have indicated that *Moringa* contains high concentrations of many essential macro- and micro-nutrients, for example, Kumssa *et al*. [6] and Olson *et al*. [7]. The use of the edible parts of *Moringa* by MGHs and for their livestock had been widely demonstrated in the present study. More than 78% of the MGHs in S. ETH and KEN had been utilising MO and MS edible parts in their diet and >71% were engaged in cultivating these species for >17 years. This level of dietary usage of *Moringa* was similar to those in Nigeria where 71% (n = 745) of the respondents in ethno-pharmacological survey reported food and nutritional utilization of MO edible parts [16]. Human dietary usage of *Moringa* spp. was mainly boiled fresh leaves, and leaf powders mixed with other dishes and as tea. Immature pods and flowers were used as vegetables by some MGHs. These modes of dietary utilization were consistent with previous reports [8, 10, 13, 23, 47].

Based on the response of >90% of the MGH heads from S. ETH, MS leaves were used in their diet on a daily basis. The qualitative big, medium and small bunch sizes reported by the respondents translate to 300 g, 250 g, and 200 g [48]. Accordingly, a MGH consuming a big bunch of MS leaves in Ethiopia can obtain 123% of the selenium (Se) daily-recommended nutrient intake (RNI) of a healthy adult man. Similarly, a MGH in Kenya consuming a big bunch of MS leaves can obtain 432% of the Se RNI for a healthy adult man [6]. Further accurate quantification of the dietary nutritional contributions of MS and MO to household nutritional security through a specifically designed dietary survey would be a valuable next step.

Edible parts of MO and MS used in peoples’ diet could play a useful role in tackling hidden hunger. Nevertheless, research findings are scanty on the bioavailability of these nutrients when ingested by humans. A few studies on bioavailability of some nutrients from *Moringa* leaves indicated variation between nutrients. For example, an *in vitro* study showed that iron bioavailability from MO leaves was very low while beta-carotene bioavailability was 100% [49] which was consistent with Nambiar and Seshadri [50]. Similarly, when MO leaves were fed to Wistar rats, >80% of the folate was bioavailable [51].

Phytochemicals in the edible parts and other tissues of these plants are reported to possess therapeutic properties to treat, for example, anaemia [49], common cold [52], diabetes [33, 34, 37, 53], eye and ear infections [37], hyperlipidaemia [54], hypertension [55, 56], leprosy [12], malaria [57], typhoid [8, 23, 58]. They also possess bactericidal and fungicidal properties [13, 59]. Recent *in vitro* research reports indicated that MO leaf extracts had cytotoxic effect on the A459 lung cancer cell lines [60] and oesophageal cancer [61]. The moringin extracted from MO leaves has been reported to have a beneficial role in preventing cancer [62]. Furthermore, MO can be used in the production of gold nanoparticles that are used in cancer therapy [63]. Some of the medicinal values of *Moringa* were stated as useful side benefits by respondents from S. ETH and KEN in the present study.

A common way of cultivating the *Moringa* trees, in both S. ETH and KEN was intercropping with other staple food crops, for example, cassava, maize and sorghum. Under such type of land use, the *Moringa* leaves shed on the soil serve as green manure to increase soil fertility and boost crop yield [12, 38, 64–70]. Some interviewees in KEN indicated that hedges of MO shrubs had been used for soil conservation.

### Off-farm benefits, challenges and opportunities

The seed oil from the *Moringa* is sought after in the soap and fragrance industry because of its ability to absorb and retain fragrances [14, 71], in the energy sector to manufacture biodiesel [52, 72–78], and for water purification as a natural coagulant [16, 20, 79–86]. Although these various uses that are derived from the *Moringa* seeds can be an off-farm opportunity to raise household incomes, lack of access to markets was one of the challenges that was faced by the MGHs. Difficulties with a reported failure of an international buyer of MO leaves by some KEN households shows the importance of secure markets which allow the producer to develop this perennial crop. Although those MGHs were disappointed because the economic gain did not materialize, this resource, for example, could still be used to fulfil the mineral nutritional requirements of their household and/or livestock especially during the dry season and at the onset of the rainy season when other vegetables and forage crops are scarce [27, 87–89]. Raising community-wide awareness on the multiple uses of *Moringa* is required to create market demand and maximize resource utilization.

Other reported challenges were diseases and pests, and parasitic plants. The diseases and pests reported by the interviewees were in agreement with documented entomological and pathological information on MO. In their review, Kotikal and Math [90] categorized insect pests associated with MO in India as defoliators, sap feeders, and bark, pod and seed borers, and have listed non-insect pests. Yusuf and Yusif [91] confirmed the presence of MO leaf feeding insect larvae (*Ulopeza phaeothoracica*) in Nigeria. *Moringa* leaves browsed and shredded by insect larva are less appealing for human dietary consumption. Furthermore, diseases, pests and parasitic plants lead to decrease in foliage biomass production and in extreme cases kill the trees. These suggest a need for pathological and entomological research efforts to identify the diseases and pests, and devise control measures that do not contravene with the dietary usage of the edible parts.

## Conclusion

Ethiopia and Kenya are among the countries where hidden hunger is widespread. The survey reported here has generated useful information on human dietary usage and other common uses of MO and MS by communities in southern Ethiopia and Kenya. The participating MGH include subsistence farmers, most likely to be affected by hidden hunger and thus benefit from a perennial, drought resilient food plant. Where *Moringa* growers in these countries use MO or MS edible parts in their diet, this is likely to contribute to better mineral nutrition of the consumers. There are differences between regions in how *Moringa* is typically used, and which parts of the plant are consumed in meals, suggesting opportunities exist to learn more from these varying uses, which could influence any extension work. This could also help to understand why some MGH in Kenya choose not to eat *Moringa*.

We found a high level of awareness of multiple roles of *Moringa* among MGHs, although as with diets, these reported uses varied between regions. The presence of MGHs with *Moringa* saplings of one year or less indicates that the cultivation of *Moringa* is expanding. We have also documented some of the potential barriers to further widening use of these species, according to current growers. These particularly relate to: increasing evidence as to the benefits of *Moringa* products; pest/disease control; and, access to secure markets.

The perennial nature, multiple uses, and resilience to drought of *Moringa* species make them a suitable target for more agro-silvicultural, nutritional, and pharmacological research. A comprehensive, integrated and multidisciplinary research effort, and links with development and extension agents are required on these multipurpose tree species to develop them not only as crops to contribute to the alleviation of hidden hunger, but to potentially develop a commodity crop that can improve some of the multifaceted socioeconomic problems in tropical and subtropical developing countries. Although the multiple uses and services that can be derived from *Moringa* spp. are opportunities, maximization of the potential benefits requires research, extension and developmental priority setting in consultation with the stakeholders to better understand their viability.

## Supporting information

S1 Appendix Information sheet for participants. (PDF)

S2 Appendix Consent form provided to the respondents prior to interview. (PDF)

S3 Appendix Questionnaire used for the survey in southern Ethiopia. (PDF)

S4 Appendix Questionnaire used for the survey in Kenya. (PDF)
