# Supplementary material for: Challenges and opportunities for Moringa growers in southern Ethiopia and Kenya
Source: PLoS One. 2017 Nov 9;12(11):e0187651. doi: 10.1371/journal.pone.0187651 (PMC5679577; doi:10.1371/journal.pone.0187651)
Supplement: S1 Appendix — (PDF) [file pone.0187651.s001.pdf]

S1 Appendix. Information sheet for participants.

"Why do people in various regions grow *Moringa* spp.?"

(Ethical Approval number)

Invitation

You are being invited to be involved in a research study; before you decide whether you want to take part, it is important for you to understand why the research is being done and what your participation will involve. Please take time to read the following information carefully and discuss it with other people if you wish. Please contact me if anything is unclear or if you would like more information. Take time to decide whether or not you wish to take part.

What is the purpose of this study?

The aim of this study is; to establish why people in various countries grow *Moringa* spp. and to assess the potential of this species as multi-nutrient dietary sources for human being.

Why I have been chosen?

You are randomly selected because you have planted *Moringa* spp. There is no specific reason other than that you are one of those who grow *Moringa* to whom the study is relevant.

What will participation involve?

You will be asked a number of questions with your general household, *Moringa* spp. planting, and the products/services you draw from this species. Your responses to the questions will be written down on a mobile phone or tablet.

What if I decide that I don't want to take part?

You are free to decide that you don't want to take part in the study and can:

1. Refuse to answer any questions that you don't want to
2. Decide to stop the interview at any time
3. Remove your consent for the data collected to be used.

Will I be paid for my time?

There is no payment for taking part in this study.

Will I be anonymous, and who will know my identity?

If you agree to take part in an interview, a Participant Number will be generated for you, and that's the only thing that will be used to identify you. Your identity will only be known by the interviewer, and will not be found in any record. Hard copy and electronic data will be stored on the University of Nottingham's Network: this will be deleted after 5 years, or if you withdraw your consent (whichever is sooner).

What do I do next?

If you'd like to be involved in this study we will interview you when we visit your *Moringa* spp. that you have planted. At the interview you'll be issued with one of these information sheets, and will be asked to sign a consent form.

Who shall I contact with any questions?

To be part of this study, or to ask any questions, then please get in contact with the Principal Investigator, Diriba Kumssa:

Email: stxdbk@nottingham.ac.uk

Tel.: +447446119038
