# Supplementary material for: Challenges and opportunities for Moringa growers in southern Ethiopia and Kenya
Source: PLoS One. 2017 Nov 9;12(11):e0187651. doi: 10.1371/journal.pone.0187651 (PMC5679577; doi:10.1371/journal.pone.0187651)
Supplement: S2 Appendix — (PDF) [file pone.0187651.s002.pdf]

S2 Appendix. Consent form provided to the respondents prior to interview.

You have been invited to take part in a research project; in order to go forward with your participation, it is necessary for you to give your consent.

By completing this form you are consenting to take part in this research project; you can withdraw your consent at any point. To withdraw your consent, please either mention that to the Interviewer during the interview or contact Mr Diriba Kumssa by email before 20 July 2015. Before signing this form, please read the following statements and indicate that you agree with them by initialling next to them.

	Initial here
I have been issued with a Participant Information Sheet	
I have been informed what the purpose of this research is, and the nature of the study	
I have been informed how the data that are collected within the research will be handled and stored.	
I have been informed that I can remove my consent at any time either during, or after the interview (up to the 20 April 2015), and that withdrawal of consent will not harm me in any way.	
I have been informed that the interview will be written down on mobile phone/tablet	

I have been informed that my anonymised quotes may be used within the reporting of this research.	
I agree to take part in this study	

Signed by………………………………………. Date…………………

Consent received by……………………………….Date…………………
