# Supplementary material for: Challenges and opportunities for Moringa growers in southern Ethiopia and Kenya
Source: PLoS One. 2017 Nov 9;12(11):e0187651. doi: 10.1371/journal.pone.0187651 (PMC5679577; doi:10.1371/journal.pone.0187651)
Supplement: S3 Appendix — (PDF) [file pone.0187651.s003.pdf]

S3 Appendix. Questionnaire used for the survey in southern Ethiopia.

Moringa spp as a multi-nutrient dietary source (on farm/ home garden) - general-2

Country

- ☐ Ethiopia
- ☐ Kenya
- ☐ India
- ☐ Malawi
- ☐ Malaysia
- ☐ Tanzania

GPS location

GPS coordinates can only be collected when outside.

latitude (x.y °) longitude (x.y °) altitude (m) accuracy (m)

Household ID

What is the gender of the household head?

- ◯ Female
- ◯ Male

Marital status

- ◯ Married
- ◯ Single
- ◯ Other

If the marital status is other, please give details

How old is the household head (years)?

How many members does the household have?

For how long have you been growing Moringa (years)?

Species

- ◯ Moringa arborea
- ◯ Moringa borziana
- ◯ Moringa concanensis
- ◯ Moringa drouhardii
- ◯ Moringa hildebrandtii
- ◯ Moringa longituba
- ◯ Moringa oleifera
- ◯ Moringa ovalifolia
- ◯ Moringa peregrina
- ◯ Moringa pygmaea
- ◯ Moringa rivae subsp. longisiliqua
- ◯ Moringa rivae subsp. rivae
- ◯ Moringa ruspoliana
- ◯ Moringa stenopetala

Why do you plant Moringa?

- [ ] Food
- [ ] Medicine
- [ ] Shade
- [ ] Ornament
- [ ] Shelterbelt
- [ ] Feed
- [ ] Green manure
- [ ] Other

Additional comment on reasons of growing Moringa

____

How do you use the different parts of Moringa tree for food?

____

How do you use the different parts of Moringa tree as medicine?

____

Moringa samples collected

- [ ] Mature leaves
- [ ] Young leaves
- [ ] Green pods
- [ ] Seeds
- [ ] Roots

Moringa tree

Moringa leaves

Moringa green pods

Moringa seed

Root samples

Soil sample
