# Supplementary material for: Challenges and opportunities for Moringa growers in southern Ethiopia and Kenya
Source: PLoS One. 2017 Nov 9;12(11):e0187651. doi: 10.1371/journal.pone.0187651 (PMC5679577; doi:10.1371/journal.pone.0187651)
Supplement: S4 Appendix — (PDF) [file pone.0187651.s004.pdf]

S4 Appendix. Questionnaire used for the survey in Kenya.

KEFRI Questionnaire

Background

Country

- ☐ Ethiopia
- ☐ Kenya

GPS Location

GPS coordinates can only be collected when outside.

latitude (x.y °)	longitude (x.y °)	altitude (m)	accuracy (m)

Date of interview

yyyy-mm-dd ____________ hh:mm ____________

Name of interviewer

- ◯ Charles Magare
- ◯ David Odee
- ◯ James Gitu

Name of Interviewee/farmer

____

Household ID

____

What is the gender of the household head?

- ◯ Female
- ◯ Male

How old is the household head (years)?

____

Marital status

- ◯ Married
- ◯ Single
- ◯ Other

If the marital status is other, please give details

What is the highest educational achievement of household head?

- ◯ Illiterate
- ◯ Elementary
- ◯ Highschool
- ◯ College/University

How many people (full time residents) live in the household/farm?

What is your ethnic group?

Land tenure

How is the land owned?

- ◯ Own
- ◯ Rental
- ◯ Communal
- ◯ Others

If Others, please specify

What is the approximate size/acreage of your farm?

- ◯ 1-3 acres
- ◯ 4-10 acres
- ◯ 11-15 acres
- ◯ 16-20 acres
- ◯ > 20 acres

Are you engaged in any other off farm income generating activities?

- ◯ Yes
- ◯ No

If 'yes', what's the activity/ profession?

- ○ Formal
- ○ Self-employed
- ○ Casual labour
- ○ Others

If 'Formal', please specify

____

If 'Others', please specify

____

What crops do you grow on the farm? (Rank in order of importance)

____

Do you keep any livestock?

- ○ Yes
- ○ No

If 'Yes', list/rank in order of importance

____

For how long have you grown Moringa (years)?

____

Why do you plant Moringa?

- ☐ Food
- ☐ Medicine
- ☐ Shade
- ☐ Ornament
- ☐ Shelterbelt
- ☐ Feed
- ☐ Green manure
- ☐ Other

What part of the tree did you use to plant?

- [ ] Seed
- [ ] Seedlings
- [ ] Branch cuttings
- [ ] Stem cuttings

Where (or from who/whom) did you source your planting material?

____

Species

- ○ Moringa oleifera
- ○ Moringa stenopetala

How do you use Moringa? Prompt with the list below if not mentioned

- [ ] Leaves
- [ ] Bark
- [ ] Twigs
- [ ] Roots/tubers
- [ ] Green pods
- [ ] Stems
- [ ] Dry pods
- [ ] Branches
- [ ] Seeds
- [ ] Flowers
- [ ] Whole plant
- [ ] Fodder for livestock
- [ ] Firewood for cooking
- [ ] Water clarification
- [ ] Agroforestry: intercrop, hedge, boundary, aesthetic, green manure, shade, etc
- [ ] Other

Description of use

____

Description of use-1

____

Description of use-2

____

Description of use-3

Description of use-4

Do you sell any of the Moringa plant parts or products?

- Yes
- No

If 'Yes', list Moringa plant parts/products, value and buyers (namely green leaves, dry leaves, greed pods, dry pods, seeds, fresh flowers, dry flowers, branches, stem roots, tubers, firewood

If 'Yes', list Moringa plant parts/products, value and buyers (namely green leaves, dry leaves, greed pods, dry pods, seeds, fresh flowers, dry flowers, branches, stem roots, tubers, firewood

Management

How often do you harvest or extract Moringa parts for use or sale?

- Monthly
- Quarterly
- Bi-annually
- Annually
- Other

If 'other', please specify

What is the estimated number of Moringa tree/saplings on the farm?

- 0-30
- 30-60
- 60-100
- > 100

Have you experienced any problems with pest, disease or livestock/wildlife damage? Moringa tree?

- Yes
- No

If yes, describe the causal agent, type and part of the tree damaged

- ☐ Leaves
- ☐ Roots/Tuber
- ☐ Stems
- ☐ Green pods
- ☐ Dry pods
- ☐ Branches
- ☐ Seeds
- ☐ Flowers

Description of the damage

____

Description of the damage

____

Description of the damage

____

If pest or disease attack, what period, season or time of the year when it most severe?

____

What intervention or control methods did you use?

____

Do you have any questions or comments about Moringa or general comments?

____

Would like to receive or know more information regarding Moringa?

- ○ Yes
- ○ No

Observations to be made by the interviewing team (planting configuration, location, regeneration, management)

____

What is the tree arrangement on the farm?

- [ ] Scattered
- [ ] Isolated
- [ ] Lines
- [ ] Woodlot

Where on the farm the trees are planted/located?

- [ ] Farm/home boundary
- [ ] Hedge
- [ ] Intercropping with other crops

Are there any signs of natural regeneration or fresh plantings?

- [ ] Young seedlings
- [ ] Saplings
- [ ] Sprouting
- [ ] None

How are the Moringa trees managed or tended?

- [ ] Coppicing
- [ ] Pruning
- [ ] Lopping
- [ ] Pollarding
- [ ] Spot weeding
- [ ] Clear weeding

Where do you get water for domestic use?

____

Do you find it necessary to cleanse or purify water for domestic use?

- ○ Yes
- ○ No

If 'Yes', how do you treat your water?

____

From what material is your house roof made?

____

From what material is your house floor made?

____

Do you have electricity in your house?

- ◯ Yes
- ◯ No

Moringa samples collected

- ☐ Mature leaves
- ☐ Young leaves
- ☐ Green pods
- ☐ Seeds
- ☐ Roots

Leaves sample ID

____

Green pod sample ID

____

Seed sample ID

____

Soil sample ID

____

Moringa tree

Moringa leaves

Moringa green pods

Moringa seed

Root samples

Soil samples

What in-the-field treatment is applied to the sample? (e.g., washing with water)

What is the approximate height of the tree? (metres)

What is the approximate diameter at breast height of the tree? (centimetres)

At what depth was the soil sample taken? (centimetres)

Estimate the distance between soil sampling point/s and the base of the Moringa tree (metres)

Thanks for completing the questionnaire!
